# Perceived poverty and health, and their roles in the poverty-health vicious cycle: a qualitative study of major stakeholders in the healthcare setting in Hong Kong

**DOI:** 10.1186/s12939-020-1127-7

**Published:** 2020-01-28

**Authors:** Gary Ka-Ki Chung, Dong Dong, Samuel Yeung-Shan Wong, Hung Wong, Roger Yat-Nork Chung

**Affiliations:** 1 The Jockey Club School of Public Health and Primary Care, The Chinese University of Hong Kong, New Territories, Hong Kong; 2 Department of Social Work, The Chinese University of Hong Kong, New Territories, Hong Kong

**Keywords:** Poverty, Health, Cycle, Perception, Qualitative, Healthcare setting, Hong Kong

## Abstract

**Background:**

Poverty and ill-health are closely inter-related. Existing studies on the poverty-health vicious cycle focus mainly on less developed countries, where the identified mechanisms linking between poverty and ill-health may not fit the situations in developed Asian regions. This study aims to qualitatively explore the perceived mechanisms and drivers of the poverty-health vicious cycle among major stakeholders in the healthcare setting in Hong Kong.

**Methods:**

Data were collected via focus group interviews with social workers (*n* = 8), chronically ill patients (*n* = 8), older adults (*n* = 6), primary care doctors (*n* = 7) and informal caregivers (*n* = 10). The transcribed data were then closely read to capture key themes using thematic analyses informed by social constructivism.

**Results:**

In this highly developed Asian setting with income inequality among the greatest in the world, the poverty-health vicious cycle operates. Material and social constraints, as a result of unequal power and opportunities, appear to play a pivotal role in creating uneven distribution of social determinants of health. The subsequent healthcare access also varies across the social ladder under the dual-track healthcare system in Hong Kong. As health deteriorates, financial hardship is often resulted in the absence of sufficient and coordinated healthcare, welfare and labour policy interventions. In addition to the mechanisms, policy drivers of the cycle were also discussed based on the respondents’ perceived understanding of the nature of poverty and its operationalization in public policies, as well as of the digressive conceptions of disease among different stakeholders.

**Conclusions:**

The poverty-health vicious cycle has remained a great challenge in Hong Kong despite its economic prosperity. To break the cycle, potential policy directions include the adoption of proportionate universalism, social integration and the strengthening of medical-social collaboration.

## Background

### Introduction

Poverty and ill-health are inter-linked. The bilateral associations between poverty and ill-health result in a vicious cycle, especially in less developed countries with inadequate healthcare and welfare support systems [1]. The classic conceptual framework on poverty-health vicious cycle proposed by Wagstaff illustrated that ill-health affects individuals’ financial status through loss of income and increased susceptibility to catastrophic healthcare cost, whereas poverty causes ill-health as the poor suffer from different kinds of health-compromising tangible and intangible deprivations [1]. Dahlgren further stressed on the financial and social consequences of ill-health in terms of increased debts, disposal of assets and thus exacerbated poverty [2]. Despite the established conceptual frameworks, studies on the poverty-health vicious cycle are mainly documented in less developed countries [3, 4], which may have limited generalizability to developed settings facing a distinct set of political, economic, cultural and social obstacles.

In addition to the mechanisms linking between poverty and ill-health, structural determinants of health inequities, in terms of socioeconomic, political and cultural contexts as illustrated in the World Health Organization (WHO) Conceptual Framework for Action on the Social Determinants of Health [5], play a significant role in generating social stratification, thus shaping the distribution of the more down-stream intermediary determinants of health. Nonetheless, these contextual environments differ substantially between developed and less developed settings. In developed settings, poverty is not simply defined by absolute minimal physiological needs but exists in relation to the social norm of a population as a form of relative deprivation of diets, amenities, and social activities. While absolute material living standards may be critical drivers to ill-health in less developed countries, poor lifestyle choices and psychological stress, as a result of relative deprivation, are expected to link more closely to the great burden of non-communicable diseases in the developed world [6]. Also, their investments on healthcare and social welfare are not comparable due to differential economic capacities and policy initiatives. Consistent with past studies in the developed regions, income distribution and social policy provisions appear to have strong impact on poverty cycles and health inequities [7]. Also, a recent review paper commissioned by the King’s Fund suggested that the National Health Service, the public healthcare system in the United Kingdom, can be better designed to tackle poverty through its impact on health, income distribution, employment and service commissioning [8]. Without strengthened healthcare and welfare systems, the “21^st^ century health-poverty trap”, as coined by Bor et al. [9], is expected to emerge and subsequently widen inequalities in health [9–11]. In view of these fundamental differences, understanding whether, how and why the poverty-health vicious cycle operates in developed settings may go beyond delineating the mechanisms but inform the inadequacies of existing policies or interventions and hence alternative strategic directions to further alleviate the vicious cycle. Given the complex dynamics of the influence of structural determinants and their interactions with the down-stream intermediary factors, it is necessary to adopt qualitative research methods for an in-depth understanding of the poverty-health vicious cycle.

An in-depth investigation in Asian settings is particularly warranted given that a pooled analysis on 24 cohort studies showed a greater association of socioeconomic status with premature death and risk of cardiovascular diseases in Asian populations when compared with that in the Western populations [12]. Hong Kong, a leading Asian economy, serves as an ideal setting for understanding the dynamics of poverty-health vicious cycle and health inequities, given its all-time high Gini Index of 0.539 in 2016 with almost 20% of Hong Kong’s households living in poverty [13, 14] as well as the greatest housing affordability issue across the globe [15]. Furthermore, as one of the first Asian populations to have experienced rapid economic and epidemiological transitions over the recent decades [16, 17], Hong Kong could also act as an exemplar to other emerging economies in Asia.

The present study aimed to collect views from multiple major stakeholders of the healthcare setting, in order to explore the perceived mechanisms and drivers of the poverty-health vicious cycle under the highly developed but socially unequal context of Hong Kong.

### Poverty and health in context

#### Poverty as a multi-dimensional concept in Hong Kong

Poverty is often measured by income and defined as those living under a threshold income level relative to the social norm of a population. Although the conventional use of household income level is simple and straightforward, such an approach focuses on the monetary conditions but omits other important aspects of poverty such as non-monetary resources and social barriers to achieving improved living standard [18–20]. According to the classic Townsend’s theory of relative deprivation, poverty is defined as lack of command over sufficient resources in terms of diets, amenities and social activities over time [21], which takes individual’s purchasing power and affordability of basic necessities into account. Local reviews on poverty in Hong Kong also revealed a low overlap between income poverty and deprivation, suggesting that the two concepts play distinct roles in identifying the most vulnerable social groups [22, 23]. In other words, the choice of approach to measuring poverty would affect the estimated size of poverty, the coverage of people in financial needs and also the eligibility for allowance entitlement or other welfare policies. Moreover, a few previous studies have demonstrated the independent role of relative deprivation as a social determinant of a range of physical and mental health outcomes beyond and above the effects of household income and other socio-economic factors [19, 20, 24]. These findings stressed the shortfalls of using only income to define poverty and predict health outcomes, since income poverty does not fully represent one’s purchasing power to basic necessities of daily lives. This multi-dimensional concept of poverty is closer than merely income poverty to the subjective understanding and perception of poverty of the respondents because they did not have a clear cut-off when they referred to the concept of poverty.

#### A situational-adaption perspective of health and disease

Standing at the other end of the poverty-health vicious cycle is “health.” Similar to other developed world regions, Hong Kong has undergone the epidemiological transition with the major disease burden shifting from acute communicable diseases to chronic non-communicable diseases since the mid-twentieth century [25]. In the past decade, cancers, pneumonia, cardiovascular diseases and cerebrovascular diseases have been the leading causes of death in Hong Kong [26], whereas mental health disorders are also a major public health concern [27]. According to the definition adopted by the WHO since 1946, health is defined as “*a state of complete physical, mental and social well-being and not merely the absence of disease or infirmity*” [28]. However, whether a person feels that he/she is “healthy or not” is not simply determined by objective standards; it is also perceived subjectively [29].

American sociologist Angelo Alonzo proposed to use a “situational-adaption perspective” to understand health and disease, and pointed out that disease should not be conceptualized solely as the physiological symptoms within the body but also as the manifestations of these symptoms when they affect a person’s experiences in everyday life situations [30]. Disease may arise when a person can no longer contain these symptoms within or adapt them to certain situations (such as work) and thus has to stay away from these daily situations, such as taking sick leave from the workplace, and seeking medical care instead of doing household work [31]. In other words, a condition may not manifest itself as a “disease” if the person’s relation to their social situational environment is not interrupted.

With reference to Alonzo’s “situational-adaption perspective” of health and disease, we first would argue that it is problematic to define health statuses and disease solely by objective diagnostic criteria, which is typical in Hong Kong, where the healthcare system is dominated by Western biomedicines. Such type of “medical model” to disease [30] overlooks the context and social conditions that the patients would have to adapt to and interact with while dealing with their diseases [32]. This parallels the adaptive health model [33], which stipulates that the optimal health and well-being of an individual are determined by person-environment interactions. According to the WHO [5], factors that affect health status come from both the macro (structural/institutional) and the micro (individual/situational) levels. More importantly, these factors are constantly interacting with and constituting each other [34].

### Research questions

Previous research on the poverty-health vicious cycle seems to have a stronger emphasis on each level (most often on the macro-level only), but pays little attention to the dynamics between the two levels. Therefore, the present focus group interview study among major stakeholders in the healthcare setting aimed to qualitatively understand their perceived mechanisms of the poverty-health vicious cycle in Hong Kong, the interactions between the macro- and micro-level factors, and to identify potential policy directions by exploring how these mechanisms are driven by the macro-level social context. In particular, there are two major research questions as follows:

1.1.1 How and why, in today’s Hong Kong, the poorer become the sicker, and vice versa?
1.1.2 What are the potential policy interventions that may disrupt the poverty-health vicious cycle in the context of Hong Kong?

## Methods

### Data collection and study population

A qualitative focus group study dedicated to exploring the potential social determinants of the poverty-health vicious cycle and health inequities was completed between September and December in 2012. Respondents were recruited via related non-governmental organizations (NGOs) and public healthcare clinics. Each 2-h focus group interview was expected to consist of 6 to 8 respondents. To compensate for possible absence, 2 to 3 respondents were additionally invited in each focus group. As probability sampling is inappropriate for qualitative research, we adopted the maximum variation sampling by deliberately interviewing a range of major stakeholders in the healthcare setting so as to maximise the diversity and representativeness of opinions [35]. In total, 5 focus group interviews of different types of stakeholders, with 39 respondents (8 social workers, 8 chronically ill patients, 6 older adults above 60 years old, 7 primary care doctors and 10 caregivers for mentally disabled children), were conducted. An additional table shows the sociodemographic characteristics of the respondents [see Additional file 1]. The focus group interviews for the chronically ill patients, older adults and primary care doctors happened in a meeting room in a public healthcare clinic building, while those for the social workers and caregivers happened in a meeting room of the corresponding NGOs’ buildings where the social workers worked and the caregivers volunteered. All the respondents provided written informed consent. A trained moderator led the discussions with the assistance of researchers responsible for note taking and observing interactions among respondents. The discussions were audio-taped, followed by verbatim transcription in Cantonese and then translation into English. The transcripts were cross-checked by team members to ensure accuracy.

### Measurement

Separate semi-structured interview guides were developed for stakeholders from different disciplines to capture relevant information and experience in their respective fields. Questions were developed based on the WHO Social Determinants of Health Framework [5]. Questions for this exploratory qualitative study were divided into three parts. First, subjects were asked about the perception and criteria of a healthy life. Second, subjects were guided to share their experience and understanding on whether and how poverty and social disadvantages affect health both before and after diseases occur, as well as whether and how ill-health in turn exacerbate poverty. Last, subjects were asked to comment on whether the poverty-health vicious cycle and associated health inequalities, if any, are inequitable (i.e., unfair), and to suggest potential causes and solutions to the issue.

### Data analysis

Thematic analysis was used to identify social determinants and dynamics of mechanisms leading to the poverty-health vicious cycle, as well as the potential causes and suggested solutions to the cycle. The transcripts were closely read to capture key ideas for preliminary coding based on a rigorous qualitative descriptive approach from a social constructivist perspective, which presumes that meaning and experience are socially produced rather than inhering within individuals, and is therefore suitable for theorising the social contexts and structural conditions based on individual’s experiences [36]. The development process and meanings of ideas were documented in memos during analysis. The transcribed texts were then categorized into manageable units for coding and sorting. A final list of key themes and codes was achieved upon consensus on the coding categories by at least two independent researchers. Data were validated through data triangulation (i.e., by checking data in transcripts and field notes), investigator triangulation (i.e., by involving two team members for coding independently), and methodological triangulation (i.e., by supplementing transcribed data with on-site observations). Excel spreadsheet was used for manual coding and management of the identified codes.

## Results

### Perceived social determinants of health: lifestyle, psychosocial impact and the healthcare system

Most of the participants agreed that unhealthy lifestyle led to poor health and that people living in poverty tended to have a less healthy lifestyle, which is, as they recognized, driven by a greater material and social constraint. Many respondents pointed out that poorer people could hardly consume healthy foods, such as fruits, vegetables and meals containing less fat or monosodium glutamate (MSG), due to limited budget. Instead, fast foods are the common affordable choices for their daily lives:

*“I have heard of some cases in our project that older patients who are receiving a very low income and relying on old age allowances cannot afford pricier meals of higher quality or less MSG.”* [Social worker_03]

*“For instance, we may have a perception that the poor cannot afford luxury foods and therefore should be at a much lower risk of hypertension or diabetes. But it is in fact the other way round. The poor may end up consuming more fast foods, like meals in McDonald’s, which are cheaper than a basic meal set.”* [Primary care doctor_07]

However, price only reveals one side of the story. The poor, as some respondents further argued, was deprived the choice and power to a healthier lifestyle:

*“When you can’t financially support your own family, what would you do? You would be powerless… You have to earn more by working for multiple jobs or for a longer time. Such a long working hour definitely harms your health and quality of life. Can you still manage to exercise? Of course not! Just go to sleep as you are already extremely tired after work.”* [Chronically ill patient_04]

*“Some working classes, such as security guards or those who work night shifts, should have a poorer control of diabetes and hypertension.”* [Social worker_03]

For most people living in poverty, if they want to earn more and strive for a better life, they have to spend more time “making money,” which however, forces them to sleep less, exercise less, or spend less time with their family. Unlike their richer counterparts, even if they have self-awareness of healthy lifestyle, the poor cannot “buy” a healthier lifestyle or exchange money for time and health. In other words, the material and social constraints limit the full potential for the disadvantaged to adopt a healthy lifestyle. As a result, their psychological health was further affected, which was highlighted throughout the discussions. Respondents had observed that the poor had more exposure to stress, which subsequently would affect other family members:

*“Imagine that the family is a circle, when you can’t make money, your wife will worry in addition to worrying about the academic performance of the children. Everything intertwines with the financial hardship… don’t you think they are all different kinds of pressures? It is indeed like a pressure cooker. It’s only a matter of time when we finally can’t stand it, right?”* [Chronically ill patient_07]

*“[Interviewer]: So, will financial constraints in turn affect our health?*

*[Informal caregivers_05]: Yes! Quarrels… financial hardship… these make us unhealthy.*

*[Informal caregivers_08]: Yes, just like my husband initiating quarrels and domestic violence. These are unhealthy indeed.”*

The next question then was about ways to resolve problems caused by unhealthy lifestyle and psychosocial distress. Most of the time, it is the healthcare system that constitutes a safety net of “caring” when the poor fall into devastating situations. However, the respondents’ encounters with the healthcare system in Hong Kong do not seem to be “relieving”; rather, their experiences demonstrated that worse ability to afford preventive care and to access medical services in a timely fashion might further strengthen the vicious cycle between poverty and health. For instance, both chronically ill patients and primary care doctors agreed that the poor have fewer choices for regular body check and preventive medical care due to material constraints, and in particular, money:

*“They want a body check but won’t actually take action since a basic body check costs them about a thousand dollars (in the private sector). And if they go to the public sector, we won’t offer similar body checks for them. As a result, disease prevention becomes so difficult for them.”* [Primary care doctor_01]

Moreover, most respondents agreed that the poor also faced fewer choices, more financial barriers and longer waiting time when seeking for outpatient healthcare on non-acute or non-critical conditions, as a result of a passive primary care system in Hong Kong.

*“If I fall short of money, can I have fair access to medical treatments that I need? That's … a big question mark.”* [Social worker_02]

*“The major difference between the private and the public health sector is that the former is fast while the latter is slow… Health system that operates as a private business has to be much faster, as it is profit-driven. As for the public healthcare system, it is slow and you have to wait. As long as you won’t die, that’s ok.”* [Chronically ill patient_02]

Nevertheless, if you are old enough (i.e., over 65 years of age) or extremely poor (i.e., living below the poverty line), outpatient medical care in the public sector will be offered to you at low cost that is highly subsidized by the government:

*“Hong Kong has … a safety net at least. For those who are very poor, they can still gain basic access to medical consultations.”* [Primary care doctor_05]

These arbitrary policy definitions of “old age” or “poverty” could make huge differences in healthcare access and financial protection among patients with similar medical needs:

*“As the welfare benefits are only offered to those aged above 65 or 70, those aged 60 like us have to pay the full price, right? That’s right. But several visits (in private clinics) may be required to treat the problem, leading to financial burden. Every time when I used the Telephone Appointment System (in the public sector), they (the operators) replied it is full, and they never advise when I should dial back to take a waiting number.”* [Older adult_03]

However, even if one is old and poor enough to get timely access to government-subsidized healthcare, that does not mean that one has the necessary and adequate access to medications he/she needs. Although a long list of medications is sponsored by the government in the formal Drug Formulary, several social workers and chronically ill patients mentioned the difficulty in accessing more effective but pricier medications. As one older patient remarked:

*“If I want to use a certain drug, I would need to purchase it myself, as some medications are not covered by the Government (public sector), or we cannot wait for the Government to include it in their drug formulary. Medications provided by the Government are most likely the cheaper ones.”* [Older adult_06]

On the contrary, doctors generally did not agree with the patient’s perception of the poor being prescribed with cheaper and low-quality medicines. They deemed such a claim a typical prejudiced impression:

*“[Primary care doctors_01]: I don’t think there is a relationship (between poverty and quality of medicines). This is just because they have… have an impression…*

*[Primary care doctors_06]: Just an impression only… (seemingly to echo [Primary care doctors_01])*

*[Primary care doctors_01]: that the wealthier always have better foods and medicines, and their doctors are more responsible.”*

If what the doctors in our interview claimed is true, then the poor patients are actually not as restrained by the inaccessibility to more expensive, higher quality medications as generally perceived by the patients themselves. Rather, they are contained within their own perceptions of the healthcare system, seeing it as something that they do not have the power to go against, and something that is rigid and unbreakable as if it is written in their fate.

### Perceived linkage between ill-health and poverty: medical expenditure, working opportunities and caregiving financial burden

The respondents generally agreed that medical expenses were the most direct cause of financial hardship for the disabled and chronically ill. Although social welfare and healthcare policies are in place, the eligibility criteria were generally deemed too stringent to protect people from financial hardship:

*“When you act up, or even become disabled or chronically ill, you need to seek and pay for healthcare and medications, which are not fully covered by the Government. So to us, especially the retired older people, that’s a burden, a financial burden.”* [Older adult_06]

*“These (medical conditions) are actually related to their (the patients’) financial situations. ‘Should I spend on better medications or daily livings?’ This is the kind of dilemma they have to deal with.”* [Social worker_04]

While the poor are more likely to be eligible for welfare allowances, financial catastrophe due to medical expenses may have an even greater impact on those who are merely above the eligibility threshold:

*“A middle class patient had to keep spending on treatments until being impoverished in order to be eligible… in other words, he/she originally didn’t need the financial support… um… only when the non-poor becomes officially poor, the system will then lend a helping hand.”* [Social worker_04]

*“They (the deprived) enjoy free healthcare services. Instead, the middle class with medical needs need to work but at the same time can hardly afford private services and time for healthcare seeking… They are actually even worse!”* [Primary care doctor_06]

On the other hand, financial security can hardly be achieved without adequate working opportunities. When being asked about the perceived mechanisms linking ill-health to poverty, our respondents pointed out that fewer working opportunities might be one of the key reasons why the disabled and the chronically ill could not rid themselves of poverty. They also argued that, it was not simply because of their working capabilities that they could not find a job; rather, the unfriendly working conditions and social stereotypes about people with special medical needs exclude them from the job market:

*“For example, some people can’t get a job not because of a lack of productivity but just a lack of ‘value’ in the job market... You won’t be hired when you have certain chronic diseases, and this affects your income and working opportunities.”* [Social woker_04]

Moreover, financial hardship is not only affecting patients themselves but also their caregivers, most of whom are family members. Extra caregiving expenditure and reduced time and capacity for work are common reasons for financial hardship among the caregivers:

*“The expenditure (for caregiving) has to be much greater than that for normal people (with no caregiving duties), but sometimes it’s hard to imagine how much more is needed.”* [Informal caregiver_04]

For many Hong Kong families, traditional Chinese belief such as filial piety and fraternal duty is still a dominant ideology guiding their everyday life and practice. Hence, the disease burden may eventually affect other family members of the patient. The example below illustrates such a rippling effect:

*“His elder sister said ‘I must take care of him and will move in with him. I can’t let my brother live alone.’ Then I asked her whether she has her own family to take care of, and she said “I have, but I can’t put all the different factors into my equation. Taking care of my brother has to be my priority now.”* [Social worker_05]

### Perceived linkage between individual and the social environment

When being asked whether broader socio-environmental or individual factors matter more in generating social inequalities in health, social workers, chronically ill patients and primary care doctors generally agreed that broader factors are more important, as the poor have limited resources, opportunities, choices and capability to overcome the influence of social environment on individual factors and health:

*“Inflation has been serious in Hong Kong but there hasn’t been a corresponding increase in salary among the poor, not even much allowances. When they can’t even afford the increasingly expensive items, their health and nutritional statuses won’t be improved.”* [Social worker_01]

*“The commodity price nowadays, when compared to that of the past, is much higher than what we can afford. Why? A large proportion of our lifetime earnings are destined to be taken away by banks, property developers, and owners.*

*([Interviewer]: That’s about social deprivation.)*

*Yes. So, people make less money in disguise, and can’t afford a decent living standard.”* [Chronically ill patient_04]

*“When having plenty of resources, including knowledge and money, that allow that certain person to have more choices in life, one can choose his/her own environment and lifestyle. But in cases where the person has less power, then he/she has less options, and the influence of social environment becomes much more important.”* [Primary care doctor_05]

On the other hand, most of the older adults in our study claimed that individual factors rather than the social environment are more important, and their rationale was based on a lower difficulty in altering individual factors than the social environment in practice. They generally supported the notion that the poor have lower bargaining power and control over broader determinants of health inequities:

*“Of course it would be nicer to receive financial support from the government. But if there is no such support, we can’t do anything about it, right? We are just humble citizens. How much can we really negotiate?”* [Older adult_03]

## Discussion

### The poverty-health vicious cycle in Hong Kong

**Fig. 1:**
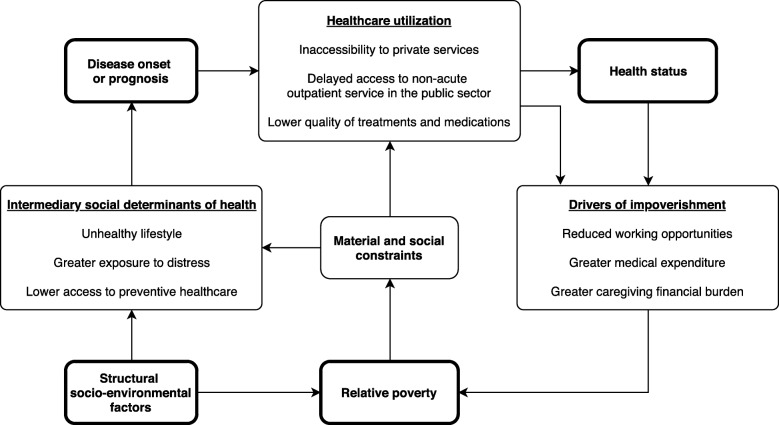
Perceived mechanisms between poverty and health

Based on the focus group interviews with the multiple major stakeholders in the healthcare setting, their perceived poverty-health cycle has been depicted in Fig. 1.

In Hong Kong, unhealthy lifestyle, increased exposure to distress and lower access to preventive care among the poor, which largely stemmed from material and social constraints, are the major factors linking poverty to disease onset or prognosis. The subsequent healthcare access also varied across the social ladder, especially in terms of access to non-acute outpatient care under the dual-track health system in Hong Kong. As health deteriorates, reduced productivity, fewer working opportunities and caregiving financial burden may result. Without sufficient policy interventions, failure to return to the workforce as well as the incurred medical and caregiving cost would lead to financial catastrophe and medical impoverishment, and thus exacerbate the financial situations of the patients and their families. The influence of structural determinants and up-stream policies on poverty and thus the poverty-health vicious cycle was generally recognised by our respondents.

### Dynamics between individual’s perceptions and the social context

#### Perceived poverty and the social environment

Our results clearly demonstrated a lack of power and opportunities among the poor to overcome and change the social environment that potentially promotes and reinforces unfavourable intermediary determinants of health. In essence, poverty is a state of disempowerment [37]. Relative social powers in a society play a central role in determining socioeconomic positions, and thus generating health inequities as a result of unequal distribution of social determinants of health [5]. Poor people with less power tend to be more constrained by the social structure, which limits their full potential of individual agency to exercise their free will even if they have a similar level of awareness and want to be healthy as the better-off do. A consequence of such an imbalance of power and opportunities would likely be a lack of affordability of material-based and social necessities in life, such as healthy diets and social activities. Although health awareness and personal choices are important in shaping individual’s health, these micro-level endeavours are deemed insufficient to tackle the poverty-health vicious cycle and health inequities in the absence of structural changes leading to redistribution of powers and resources towards the disadvantaged groups [5].

Also, by investigating various major stakeholders’ perceptions on poverty, we observed a mismatch between the official poverty definition and the actual situation of the poor and the socially disadvantaged. Given that poverty is commonly operationalized by an arbitrary threshold in terms of income or asset defined by policy makers, ill-defined poverty line that does not really reflect the actual situation in a society could exacerbate the poverty-health vicious cycle. As this arbitrary poverty line largely determines the eligibility for social allowances, its failure in reflecting the context of poverty could lead to inaccessibility to health-promoting amenities and healthcare services among the disadvantaged.

#### Perceived health and the social environment

It is a common phenomenon that our respondents put stronger emphasis on their individual health-related experiences after disease onset but less on the experiences before onset, probably because these after-onset experiences directly affect the quality of their daily life and thus may constitute their conception of situational diseases [30]. As mentioned earlier, one’s conception of health and disease depends not only on the physiological symptoms but also, or more importantly, on whether people can adapt to the difficulties resulting from these symptoms to avoid interruptions to their normal daily lives. This may also explain why our respondents emphasized less on factors leading to disease onset, as their experiences before disease onset may have a smaller impact on the quality of their daily life.

It is worth noting that the major conflicting point of view between primary care doctors and the other stakeholders lies in whether the poor had a lower quality of medications and treatments. While the service users find access to quality medications difficult due to financial constraints and insufficient sponsoring from the Government, primary care doctors generally support that prescriptions in the public sector are appropriate and could fairly meet the healthcare needs of poorer patients. The conflicting opinions suggest a disagreement between doctors’ diagnostic logic and patients’ expectation on healthcare delivery. Nonetheless, primary care doctors could hardly notice these potential clashes as service users tend to react silently and avoid direct confrontations [31], especially under the information and power asymmetry between doctors and patients. In light of it, social workers, especially those being trained in the medical field, could serve as a bridge for establishing better communication between doctors and patients [38]. Among all stakeholders, social workers demonstrated a more comprehensive understanding on patients’ experiences and the potential mechanisms between poverty and health, as the nature of their occupation tends to be more concerned about the influence of social environment on individual behaviours and health among the disadvantaged patients. Therefore, in addition to the clinical treatments prescribed by doctors, social workers could play a role in advising the situational treatments which aim to create the environment and provide activities appropriate to the patient’s emotional and interpersonal needs [31]. The assistance of social workers can enhance patient-centred care and help with recognizing the situational nature of disease during the medical consultation stage.

### Driving forces of the poverty-health vicious cycle at the macro-level

By comparing the themes generated from the focus groups and findings from previous studies, three main driving forces at the macro-level that contribute to the poverty-health vicious cycle in Hong Kong could be identified.

#### Economic factor: wealth inequality

While Hong Kong is among the leading economies in the world, the extent of income inequality, as mentioned earlier, also ranked among the top [13]. The high relative poverty rate in this developed setting suggests that poverty exists in relation to the living standards that are customary in a population [39]. The disproportionate distribution of power and opportunities is particularly apparent in regions with severe wealth inequality, a common phenomenon in both Hong Kong and also in most developed regions of the world. As a result of social stratification, the disadvantaged are generally more prone and vulnerable to a range of health-compromising intermediary factors and thus to adverse health outcomes [5].

Under the context of great wealth inequality, the rising commodity prices of healthy foods over the recent decades could disproportionately affect the disadvantaged groups. Since the 1990s, there has been a significant increase in prices of fruits and vegetables at an average annual rate of 2–3% in high-income and emerging economies; meanwhile, substantial price falls for a range of processed foods have been observed [40]. As healthier choices became increasingly difficult to afford, the disadvantaged tend to purchase and consume cheaper but less healthy foods, and thus have a greater disease risk [41, 42]. Consistently, a recent systematic review on socioeconomic inequalities in dietary intakes in Europe also provided solid evidence on a considerably lower consumption of fruits and vegetables among the disadvantaged [41]. In addition to material constraints, “time poverty” (i.e., having little time for leisure activities outside of work) was found to be a critical barrier to regular physical activity engagement and adequate sleep [43]. Long working hours, often associated with less skilled elementary workers, tend to create tensions between work and health investments, and thus may displace other health-promoting behaviours [44].

These material and social constraints also create tremendous psychosocial stressors in daily living, and the disadvantaged are often more susceptible to stress in response to stressful life events beyond their capacity and control [45]. Previous research suggested that the level of self-perceived stress could substantially explain the socioeconomic inequalities in health [46]. Under the context of great wealth inequality in Hong Kong, uneven distribution of income and resources may have significant impact on mental well-being above and beyond the effect of absolute deprivation per se [47]. This mechanism is further supported by a previous work on an inverse association between subjective feeling of personal relative deprivation and mental well-being via upward social comparison [48].

#### Health care: dual-track health system and inaccessibility of the poor

The health consequences under severe wealth inequality could also be exacerbated by the dual-track health system in Hong Kong, due to a passive primary and secondary care, as well as the imbalance of demand and capacity of tertiary care between the public and private health sectors. While inpatient care is predominantly supported by general taxation and 90% of inpatient care is publicly funded, only 30% of outpatient care is publicly funded under this mixed system in Hong Kong [49]. Private services, accounting for the majority of outpatient care, are largely paid out-of-pocket unless being covered by medical insurance or employment fringe benefits among the better-off [50].

A comparative study in Asia comparing modes of healthcare delivery in achieving equity in healthcare utilization showed that the utilization patterns in Hong Kong appear to be more pro-rich under the dual-track health system when compared to the systems in Taiwan and South Korea [49]. Despite a universal health coverage and the equality, non-refusal principle of the public sector in Hong Kong that “no one will be denied adequate medical care due to lack of means” [51], low-income older adults, who tend to have greater healthcare needs, were found to have a significantly lower total healthcare utilization than their wealthier counterparts [52, 53]. Despite a few free or subsidized screening programmes for high-risk groups recently launched by the Government, the Inverse Care Law [54] also operates in the secondary preventive care which is largely covered by the private market in Hong Kong. A local randomized controlled trial showed a significantly lower uptake of diabetic retinopathy screening among diabetic patients when a small co-payment is applied [55], whereas another local study reported that lower socioeconomic status was associated with a delayed detection of breast cancer [56]. Therefore, the disadvantaged tend to be excluded from the private sector which provides timely first-contact but more costly outpatient care as well as follow-up consultations for disease management [57].

Although a fast track is offered to urgent and more severe cases in the public sector, its limited capacity relative to the demand would inevitably lead to delay or even forgoing of medical consultations among the disadvantaged [6]. In fact, up to 8.4% of a cross-sectional general Hong Kong population sample reported that they did not seek medical care due to lack of financial means during the past year [57]. To alleviate the burden in the public sector, the Government has recently initiated public-private partnerships (PPP) so as to ensure efficient use of existing healthcare resources by subsidizing the worse-off to purchase private services [58]. While a few of the PPP programmes are receiving increasing commitment from private service providers and patients in the public sector, the inadequate technical support, compartmentalized care, and involvement of co-payment hinder the success and sustainability of these initiatives [58].

#### Social policy: safety net for financial catastrophe and medical impoverishment

A large out-of-pocket payment may incur a very high expense out of the financial resources of the patients, and therefore lead to financial hardship, including financial catastrophe and medical impoverishment. According to the WHO [59], financial catastrophe is defined as health expenditure above or equal to 40% of a household’s non-subsistence income. The high percentage spending on health could create tension and take away resources for meeting other basic needs of a household. Moreover, a more serious consequence of large out-of-pocket payment would be medical impoverishment, pushing patients below the official poverty line [59]. This phenomenon at household level has been comprehensively illustrated by Dahlgren [2] as a medical poverty trap. Sick people are pushed into poverty via increased medical cost on treatments and medications as well as reduced income due to lowered productivity, whereas the resulted impoverishment further hinders healthcare access and therefore exacerbates ill-health.

Various social welfare policies have been in place in Hong Kong. Apart from the general financial support to low-income families via the Comprehensive Social Security Assistance (CSSA) Scheme [60], the Samaritan Fund and the Community Care Fund [61] have been established to specifically provide financial assistance to needy patients for expensive treatments and medications which are not covered by the standard fees and charges in public hospitals and clinics. Nonetheless, strict eligibility criteria based on means-test limits their effectiveness in breaking the medical poverty trap. Taking the Samaritan Fund for non-drug items as an example, patients are excluded from assistance if their household, including all core family members, earn more than the median domestic household income of corresponding household size [61]. Even for those who passed the income test, only patients whose household assets are twice or less the cost of the medical items concerned would receive full assistance, whereas a sliding scale of partial contribution is applied to those with household assets two to three times as high the cost of required items [61]. In other words, patients need to be poor enough to benefit from the financial assistance, while the less poor, albeit patients who may suffer from financial catastrophe, may need to be pushed further towards poverty in order to be eligible for greater financial support and protection.

### Policy directions to tackle the poverty-health vicious cycle in Hong Kong

Several types of policy interventions regarding the macro-economic, healthcare, social welfare and labour systems have been discussed in the focus groups interviews. Although the up-stream policies such as progressive taxation and strengthening of the primary care system are important to broadly tackle poverty and disease from the outset, additional interventions in the social welfare and labour systems should also be in place to specifically address the needs of those falling into the poverty-health vicious cycle. Provision of social safety net to needy patients is one of the most common interventions to address poverty; nonetheless, whether it is effective in tackling the poverty-health vicious cycle depends on its stringency of the eligibility criteria. Currently, the social welfare schemes in Hong Kong focus on those suffering from severe financial hardship. Provided that a prejudiced attitude towards social welfare has long been rife since the colonial era of Hong Kong, the Government has devised the healthcare policies based on economic and political considerations but rarely on social reasons even after the handover of Hong Kong to Chinese sovereignty in 1997 [62]. With a lack of social philosophy in the healthcare planning in Hong Kong, the existing schemes targeting those with severe treatment burden, such as the Samaritan Fund and the Community Care Fund, could hardly protect patients from financial hardship and the medical poverty trap.

As inequalities in health and healthcare access do not only affect the worst-off but exist in a gradient [37, 63], Sir Michael Marmot suggested that we could adopt *“universalist policies that include everyone, but effort has to be proportionate to need”* [64], an approach termed the proportionate universalism. With this approach, not only would the worst-off patients be fully subsidized for medications and treatments, but all other patients could also be covered to varying extent based on both their financial affordability and medical needs. Nonetheless, the existing fragmented governmental structure with two separate Bureaus responsible for healthcare and social welfare in Hong Kong often hinders an efficient and flexible financing on social welfare schemes related to healthcare. To achieve proportionate universalism for financial assistance to the needy patients in Hong Kong, a better medical-social sectoral collaboration has to be established.

In addition to the social welfare policies, appropriate labour policies for patients at their productive age would be another key to tackling the poverty-health vicious cycle. While the Government did incentivise employers to hire workers with chronic disease or disability, the unfriendly working conditions and long working hours may reduce their readiness for work. Establishing disability-friendly working environment and promoting flexible working hours for eligible employees with special needs are essential to support their self-reliance. Public education on eliminating social stereotypes about the capability and productivity of people with special medical needs may also be crucial to facilitate the mainstreaming of disability and other chronic diseases in Hong Kong.

The key idea of these welfare policies for patients should be to encourage employment and to promote their self-reliance. We believe that a greater investment on social welfare to needy patients under the principle of proportionate universalism could not only unleash the potential of workforce of both patients and caregivers for economic development but could also substantially improve their quality of life when they can lead a sustainable life and no longer rely solely on social allowances. Furthermore, considering the situational nature of disease, securing resources to help patients re-integrate into normal everyday life situations for work and social activities would also improve their perceived health status and sense of well-being.

### Strengths and limitations

This is the first qualitative study on the perceived inter-relationship between poverty and ill-health among a range of major stakeholders in the healthcare setting in Hong Kong. Despite having only five focus groups, our maximum variation sampling approach ensures a comprehensive data collection from different perspectives, and also enables understanding on how different stakeholders view the same issue [35]. Data saturation has been reached as no more new ideas were emerged across focus group interviews. However, there are several caveats. Although the moderator has been well-trained while the respondents were recruited from multiple sources, the results may be biased due to the use of semi-structured interview guide and voluntary participation. Also, for the caregiver group in particular, their experience on taking care of chronically, mentally disabled children may not fully represent the experience on taking care of middle-aged or older adults with chronic diseases; nevertheless, as mentally disabled children often lack self-care ability, their experience may mimic more severe cases in adults with cognitive and physical impairments. Last, while our proposed policy directions are of potential benefits, context-specific cost-effectiveness or cost-utility analyses are warranted for feasibility and sustainability assessments before these policies can be put into practice.

## Conclusions

The poverty-health vicious cycle has remained a great challenge in Hong Kong despite its economic prosperity. The shortfall of the objective definitions of poverty and health in reflecting the actual context and perceived understandings among the major stakeholders in the healthcare setting appears to contribute to trapping people in the cycle. To protect patients with chronic diseases or disability from financial hardship (including financial catastrophe and impoverishment) and thus to disrupt the poverty-health vicious cycle, the concept of proportionate universalism is proposed to be incorporated into social welfare policies. Also, welfare policies can be designed to give more incentive to the patients and their caregivers for self-reliance and re-integration into normal work and social life in our society. In the long run, a strong medical-social sectoral collaboration has to be established in order to successfully implement the suggested recommendations via a better coordination of the healthcare, welfare and labor policies.

## Supplementary information

**Additional file 1.** Basic characteristics of respondents in focus group interviews. This table presents the sociodemographic characteristics of the respondents in each of the focus group interviews.

## Data Availability

The datasets used and/or analyzed during the current study are available from the corresponding author on reasonable request.

## Abbreviations

CSSA: Comprehensive Social Security Assistance
MSG: Monosodium glutamate
NGOs: Non-governmental organizations
WHO: World Health Organization
