# Supplementary material for: Perceived poverty and health, and their roles in the poverty-health vicious cycle: a qualitative study of major stakeholders in the healthcare setting in Hong Kong
Source: Int J Equity Health. 2020 Jan 28;19:13. doi: 10.1186/s12939-020-1127-7 (PMC6986077; doi:10.1186/s12939-020-1127-7)
Supplement: Supplementary file 1 — Additional file 1. Basic characteristics of respondents in focus group interviews. This table presents the sociodemographic characteristics of the respondents in each of the focus group interviews. [file 12939_2020_1127_MOESM1_ESM.pdf]

Additional Table 1. Basic characteristics of respondents in focus group interviews

	Social workers (n=8)	Chronically-ill patients (n=8)	Older adults (n=6)	Primary care doctors (n=7)	Informal caregivers (n=10)
	n (%)	n (%)	n (%)	n (%)	n (%)
Age group					
Below 30 years	4 (50.0%)	0 (0.0%)	0 (0.0%)	0 (0.0%)	0 (0.0%)
30-39 years	1 (12.5%)	0 (0.0%)	0 (0.0%)	7 (100.0%)	1 (10.0%)
40-49 years	2 (25.0%)	1 (12.5%)	0 (0.0%)	0 (0.0%)	5 (50.0%)
50-59 years	0 (0.0%)	4 (50.0%)	0 (0.0%)	0 (0.0%)	2 (20.0%)
60-69 years	0 (0.0%)	3 (37.5%)	4 (66.7%)	0 (0.0%)	1 (10.0%)
70 years or above	0 (0.0%)	0 (0.0%)	2 (33.3%)	0 (0.0%)	0 (0.0%)
Missing	1 (12.5%)	0 (0.0%)	0 (0.0%)	0 (0.0%)	1 (10.0%)
Sex					
Female	4 (50.0%)	4 (50.0%)	2 (33.3%)	3 (42.9%)	9 (100.0%)
Male	4 (50.0%)	4 (50.0%)	4 (66.7%)	4 (57.1%)	0 (0.0%)
Missing	0 (0.0%)	0 (0.0%)	0 (0.0%)	0 (0.0%)	1 (10.0%)
Education level					
Primary level or below	0 (0.0%)	5 (62.5%)	4 (66.7%)	0 (0.0%)	4 (40.0%)
Seconday level	0 (0.0%)	3 (37.5%)	1 (16.7%)	0 (0.0%)	4 (40.0%)
Tertiary level	8 (100.0%)	0 (0.0%)	1 (16.7%)	7 (100.0%)	1 (10.0%)
Missing	0 (0.0%)	0 (0.0%)	0 (0.0%)	0 (0.0%)	1 (10.0%)
Economic activity status					
Employee	8 (100.0%)	0 (0.0%)	1 (16.7%)	7 (100.0%)	0 (0.0%)
Self-employed	0 (0.0%)	0 (0.0%)	0 (0.0%)	0 (0.0%)	1 (10.0%)
Non-employed	0 (0.0%)	2 (25.0%)	0 (0.0%)	0 (0.0%)	0 (0.0%)
Retired	0 (0.0%)	2 (25.0%)	4 (66.7%)	0 (0.0%)	0 (0.0%)
Homemaker	0 (0.0%)	4 (50.0%)	1 (16.7%)	0 (0.0%)	8 (80.0%)
Missing	0 (0.0%)	0 (0.0%)	0 (0.0%)	0 (0.0%)	1 (10.0%)
